# Clinical effect of chlorhexidine and sodium fluoride on corrosion behavior and surface topography of nitinol orthodontic archwires

**DOI:** 10.1186/s12903-024-04289-4

**Published:** 2024-05-14

**Authors:** Osama Gamil Abd El Gawad Farrag, Nabeel El-Desouky Abou Shamaa, Walaa Elsayed Elgameay, Dalia A. Bayoumi

**Affiliations:** 1 Department of Orthodontic, Faculty of Dentistry, Suez Canal University, Ismaillia, Egypt; 2 Department of Orthodontic, Faculty of Dentistry, Sinai University, Qantara Branch, Ismailia, Egypt; 3 Department of Dental Biomaterials, Faculty of Dentistry, Suez Canal University, Ismaillia, Egypt

**Keywords:** Corrosion, Mouthwash, Chlorhexidine, Sodium Fluoride, Nickel Titanium

## Abstract

**Background:**

Alterations in the mechanical properties of the materials utilized in orthodontic appliances could affect the working properties of the appliances, thereby affecting clinical progress and outcome. Numerous studies have confirmed the correlation between alloy corrosion and raised surface roughness, which has a direct impact on the working characteristics of orthodontic archwires.

**Methods:**

Thirty nickel-titanium (NiTi) orthodontic archwires were utilized in this study. Patients were randomly selected and allocated into three groups according to the randomization plan; (The control group): subjects practiced regular oral hygiene; (The fluoride group): subjects used fluoride for intensive prophylaxis; (The chlorhexidine group): subjects used chlorhexidine. Representative samples were evaluated by SEM, and then SEM images with high resolution were examined using Image J software to determine the surface roughness and obtain the results for further statistical analysis.

**Results:**

Our findings indicated a significant difference was found between the three groups regarding the anterior and posterior parts between the control and the two other groups and a non-significant difference between NaF and CHX groups. Overall, the *p*-value for group comparisons was 0.000 for both parts, indicating a highly significant difference especially between the control and NaF groups.

**Conclusion:**

Mouthwashes containing sodium fluoride demonstrated more significant surface alterations than the control and CHX groups and should be prescribed in accordance with orthodontic materials to reduce side effects.

## Background and introduction

The management of dental biofilm poses a significant prophylaxis challenge in orthodontics to prevent the commonly associated tooth structure damage and gingival inflammation. Maintaining sufficient oral hygiene is the usual advice for evading these adverse effects [1].

All orthodontic treatments have increased the risk of plaque accumulation, causing damage to the tooth surface and surrounding soft tissues. Therefore, various preventive measures included the regular use of fluorides and antiseptics through orthodontic treatment [2].

In addition, antiseptics in the form of mouthwashes produced an antimicrobial effect by controlling plaque deposits, microbial reduction, gingival inflammation and healing stimulation, whereas those containing fluorides were highly effective as cariostatic agents [3]. Fluoride and antiseptic use resulted in some corrosion of dental alloys, as was mostly proved under in vitro conditions [4].

Chlorhexidine (CHX) and Sodium Fluoride (NaF) are two important chemical agents used in dentistry. Chlorhexidine is a broad-spectrum antimicrobial agent that is commonly used in oral healthcare for its antiplaque and antigingivitis effects. It is available in various forms, including mouthwash, gels, and dentifrices, and is often used as an adjunct to mechanical plaque control. NaF, on the other hand, is a fluoride compound that is widely used in oral healthcare products, including toothpaste and mouthwash, for its caries-preventive effects [5].

In orthodontic patients, maintaining good oral hygiene can be challenging due to the presence of brackets and wires, which can make cleaning difficult. This can lead to an increased risk of dental caries and gingivitis. To address this issue, CHX and NaF are often used together to improve oral hygiene and reduce the risk of dental caries and gingivitis [5].

A study found that adding a CHX0.06%/NaF0.05% combined mouth rinse to the daily oral hygiene regimen of orthodontic patients significantly improved oral hygiene status. The study also found that adding NaF to CHX mouthrinse had no adverse effect and generally, not all significantly, improved results in comparison with CHX and NaF alone. Furthermore, the effect of the combined CHX/NaF mouthrinse on Lactobacilli was remarkable [5].

However, it is important to note that chemical agents such as mouthrinses are not substitutes for thorough brushing and flossing, but they should be used as adjuncts. Therefore, it is essential to maintain good oral hygiene practices, including brushing and flossing, to ensure optimal oral health [5].

Proper biomechanics is one of the most crucial issues producing efficient orthodontic treatment. Altering the mechanical properties of the materials included in orthodontic appliances could affect the functional properties of appliances, thereby influencing clinical progress and outcome. Numerous studies have confirmed the correlation between alloy corrosion and raised surface roughness, which has a direct impact on the working characteristics of orthodontic archwires [6].

For clinical applications, it was important to determine and evaluate the corrosion resistance of nickel-titanium (NiTi) archwires in more detail to establish a correlation between surface characterization and corrosion resistance that have been conducted in vitro; and in vivo studies are expected to confirm those findings. Consequently, it was necessary to examine in vivo the impact of antiseptics (various mouthwashes) and fluorides on the corrosion performance and surface topography variations of nickel-titanium orthodontic archwires.

## Methods

### Sample size calculation

Sample size was calculated according (**Arkin., 1984)** and (**Belasic et al., 2021)** using Eqs [7, 8].:

$$n = {({Z_\alpha })^2} * {\raise0.7ex\hbox{${{{(S)}^2}}$} \!\mathord{\left/{\vphantom {{{{(S)}^2}} {{{(d)}^2}}}}\right.\kern-\nulldelimiterspace}\!\lower0.7ex\hbox{${{{(d)}^2}}$}}$$

The sample size was calculated using a computer program SPSS software for windows version 22.0 (Statistical Package for Social Science, Armonk, NY: IBM Corp), G Power version 3.1.9 based on a previous study [8]. For the present study, the power sample size was more than 80%, the significance level was 0.05, the confidence interval was 95%, and the actual power was 95.40%. To compensate for probable failure and boost the validity of the findings, the sample size was expanded to thirty archwire.

### Collection of samples

This study was conducted on thirty orthodontic archwires selected from the Department of Orthodontics Outpatient Clinic, Faculty of Dentistry, Suez Canal University, based on the inclusion and exclusion criteria: **The inclusion criteria** included patients receiving orthodontic treatment, their ages were between 18 and 30 years, both sexes, have a non-extraction approach and good oral hygiene according to oral hygiene index. **The exclusion criteria** included patients with medical conditions (such as diabetes mellitus, epilepsy, and hemophilia), periodontal diseases, women who were breastfeeding or pregnant, patients with oral habits, and smokers. Informed consent for using their medical records was given to all patients to be used in research purposes.

### Randomization

The selected patients were randomly allocated into three groups according to an online-generated randomization plan [9]. The study groups were divided into the following categories: Group 1: saliva group (control group) subjects practiced regular oral hygiene. Group 2: (fluoride group) subjects used fluoride for intensive prophylaxis. Group 3: (chlorhexidine group) subjects used chlorhexidine.

### Material preparation

Mouthwashes were manufactured with pure concentration at the Faculty of Science, Suez Canal University, Ismailia, Egypt; in order to eliminate the effect of other ingredients on corrosion behavior and guarantee the accuracy of the results.

**The study’s materials, their composition, and suppliers**:

- Rectangular nickel-titanium orthodontic archwires (0.016 × 0.022;” composition: 54.9% Ni, 44.9% Ti, 0.2% Cr); manufacturer: American Orthodontics, Sheboygan, USA.
- Chlorhexidine mouthwash (composition: chlorhexidine gluconate 0.12%), Manufacturing: Faculty of Science, Suez Canal University, Ismailia, Egypt.
- Fluoride mouthwash (composition: sodium Fluoride 0.2%), Faculty of Science, Suez Canal University, Ismailia, Egypt.

## Methodology

The following procedure was followed for all patients included in this study:

### Procedure

1. The orthodontic nickel-titanium archwire was investigated after six months of leveling and alignment.
2. In the lower arch, orthodontic archwires were used as the following, 0.016 × 0.022 rectangular nickel-titanium orthodontic archwires, in which metallic orthodontic brackets were used.
3. Each participant was instructed to brush his teeth three times daily and use 10 ml of mouthwash twice daily for 30 days.
4. All patients were instructed to uniform the manual brushing method (Charter’s technique, three times daily for 2 min) [10].
5. The first group (control group) (saliva group) Gp1: included participants who exhibit usual oral hygiene with a toothbrush and toothpaste including a low fluoride concentration. During the entire one-month study period, this protocol was commonly recommended to orthodontic patients.
6. The second and third groups (fluoride and chlorhexidine groups) utilized adjunctive oral hygiene products for one month.
7. The second group (fluoride group) was advised to brush their teeth three times daily and used 0.2% sodium fluoride twice daily for one month.
8. The third group (chlorhexidine group) was advised to brush their teeth three times daily and used mouthwash with 0.12% chlorhexidine digluconate twice daily for one month.

### Preparation of the specimens before analysis

a After removal, the orthodontic archwires were washed thoroughly under running water to remove any saliva contamination and then gently cleaned carefully with gauze, to remove any attached debris.
b After cleaning, the orthodontic archwires were adequately stored in individually color-coded plastic containers with distilled water at room temperature until the time of analysis, which plastic containers with black-covered containers for the control group, white-covered containers for the fluoride group, and pink-covered containers for the chlorhexidine group.
c Orthodontic archwires were adequately kept in distilled water at room temperature till the time of analysis to ensure the accuracy of the results, as exposing the archwires to oxygen air resulted in increased corrosion rates, the formation of localized corrosion attacks, and the accumulation of porous corrosion products [11].

### Preparation of the specimens for analysis

a After removal, the orthodontic archwires were cut into three pieces, the first from the anterior part between braces of two central incisors and two from both sides of the posterior parts mesial to buccal tubes and bands.
b Representative samples of all archwires were selected for SEM evaluation (SEM-QUANTA FEG-250) to assess the surface homogeneity with various media in comparison to as received wires.
c The orthodontic archwire pieces were placed on a microscopic slice for analysis.
d The orthodontic archwire pieces were fixed on aluminum stubs with standard diameters using carbon double sticky tape. The samples were metallic and did not require coating as it is easily conducted with the use of SEM electrons.
e Each sample was examined by SEM at 30 kV accelerating voltage using backscattered electron (BSE) imaging mode with 500x magnification and 3000x magnification.
f Each group’s corrosion behavior and surface topography were then determined by SEM images with high resolution using Image J 1.53t software developed by Wayne Rasband and contributors. National Institutes of Health. USA (Java 1.8.0_345).

### Scanning electron microscopic evaluation (SEM)

Before examining corrosion behavior and surface topography, the samples were scanned with SEM (FEI Company, Eindhoven, Netherlands). Representative sections were recorded at a magnification of 500x and 3000x for the archwires. The images of the most representative area were stored in digital form. The high-resolution SEM images were analyzed using Image J 1.53t software to inspect the surface roughness and quantify the results for further statistical analysis.

### Statistical analysis

The statistical analysis was performed by using Statistical Package for Social Science **(SPSS)** software. Data collection, revision, and tabulation were done utilizing Microsoft Excel 2016. Data were subjected to outliers’ detections and normality testing using Shapiro-Wilk.

Statistical analysis was performed utilizing the ANOVA test (one way), supported by the post hoc test (Tukey) to determine the effect of wire kind on the arithmetic mean roughness (Ra) within the same group (intragroup comparison) and comparison of the mean of Ra in the three groups at the same type of wire (intergroup comparison). *P*-value ≤ 0.05 was confirmed statistically significant (95% significance level), and *p* ≤ 0.001 was considered highly statistically significant (99% significance level).

## Results

### Scanning electron microscope (SEM) results

**Fig. 1:**
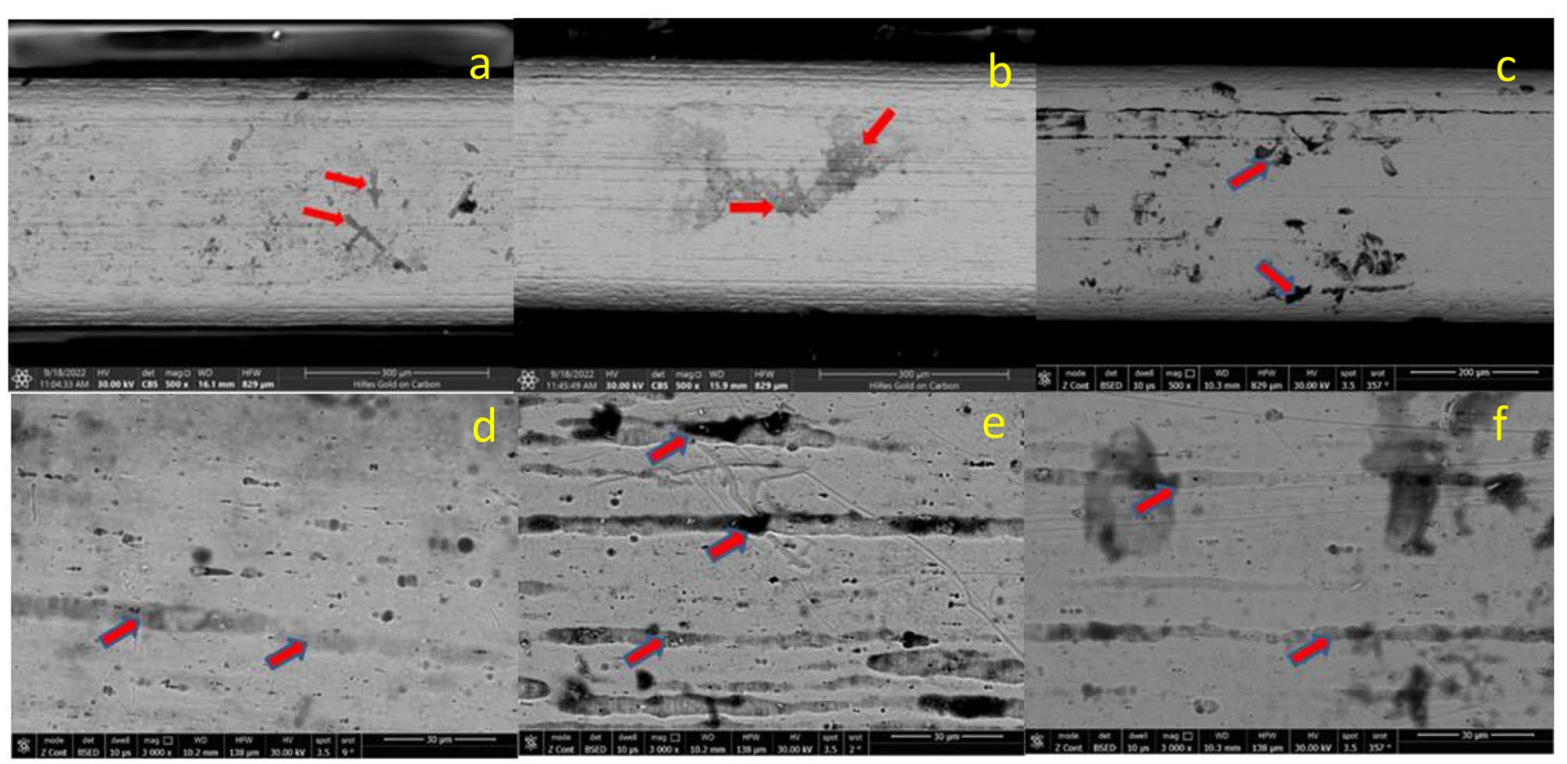
Representative SEM microphotograph (magnification 500x) for NiTi archwires (**a**) control, (**b**) NaF & (**c**) CHX. Representative SEM microphotograph (magnification 3000x) for NiTi archwires (**d**) control, (**e**) NaF & (**f**) CHX (The red arrows point to the typical defects).

Lower-resolution SEM images of NiTi archwires showed that the surface roughness in the NaF group was greater than that in the CHX group. The control groups demonstrated the lowest levels of surface roughness and corrosion defects (Fig. 1).

Long fissures, scratches, corrosion pits, and pores were visible in higher resolution SEM images of NiTi archwire (Fig. 1). NaF groups demonstrated more and deeper pits and fissures than CHX groups. NiTi archwires with control groups exhibited the fewest corrosion defects.

### Images J software results

Software analysis of the SEM images was performed for extracting surface profiles and reconstructing a 3D model. Roughness analysis was performed by calculating the roughness arithmetic average of the absolute values of the profile height deviations from the mean line. Therefore, roughness arithmetic (Ra) is the mean of a collection of micrometer-based values of surface peaks and valleys.

### A. comparison of the mean of Ra in the three groups

**Table 1:** Comparison between surface roughness of anterior and posterior parts for NiTi archwires in the three groups

Ni/Ti wire	Anterior	Posterior
Control	35.48 ± 1.43^**C**^	30.53 ± 7.63^**B**^
NaF	56.6 ± 8.03^**A**^	46.92 ± 1.44^**A**^
CHx	47.92 ± 2.51^**B**^	45.89 ± 2.71^**A**^
P-value**	**0.000** ^**HS**^	**0.000** ^**HS**^

NB: S = statistically significant (P-value ≤ 0.05), HS = Highly significant (P-value ≤ 0.001), NS = non-significant (p-value > 0.05) ** P-value for Inter-group comparison (Control vs. NaF vs. CHX). (ANOVA), Capital letters for inter-group comparison and the means with the same superscript letter are statistically non-significant (p-value > 0.05). (Tukey post hoc test)

## Discussion

Maintaining good oral hygiene in patients wearing fixed appliances is a challenge for orthodontics. Orthodontic treatment involves the application of dental archwires and attachments for tooth movement. which complicates the self-cleansing mechanisms and the saliva rinsing effect. For this reason, orthodontic patients are more prone to gingivitis and enamel decalcification. Therefore, to reduce these potential risks, mouthwash application along with brushing are mandatory. Other possible ways of antimicrobial reactions can delve into various avenues beyond traditional antibiotics. One promising approach is the use of coatings, like chitosan coatings, which have shown effectiveness in dentistry by killing fungal cells and inhibiting biofilm formation on dental biomaterials. These coatings offer a natural and biocompatible solution for combating microbial growth in dental applications. Additionally, exploring natural materials in green dentistry, such as organic toothpaste formulations, presents an eco-friendly alternative that aligns with sustainable practices in oral care [12].

Moreover, the use of Chlorhexidine and fluorides in dentistry plays a significant role in antimicrobial strategies. Chlorhexidine is a common antiseptic with broad-spectrum antimicrobial properties, widely used in oral care products for its effectiveness against bacteria and fungi. On the other hand, fluorides are essential for preventing tooth decay and maintaining oral health, showcasing antimicrobial benefits by inhibiting the growth of bacteria that cause cavities. By considering these diverse approaches, from innovative coatings like chitosan to the established use of CLX and fluorides, the field of antimicrobial reactions in dentistry can leverage a combination of natural materials, green practices, and traditional agents to enhance oral health outcomes [13].

Considering the shortcoming of clinical studies of the effects of different mouthwashes on orthodontic appliances, the purpose of this study was made to evaluate the clinical effect of chlorhexidine and sodium fluoride on surface topography and corrosion of orthodontic archwires.

- To assess the surface structure analysis of archwires, several methods can be employed. The search results present various techniques for evaluating the surface roughness and composition of orthodontic archwires.

Scanning electron microscopy (SEM): SEM is used to assess the micromorphological characteristics of the archwires. This technique allows for visual evaluation of surface irregularities and can be used to determine the surface characteristics of the wires. SEM can be equipped with an energy-dispersive X-ray spectrophotometer (EDS) to analyze the chemical composition of the wire surface. Surface profilometry: This method is performed using a contact stylus profilometer to analyze the surface roughness of the wires. The profilometer is equipped with a metal vertically movable tip that accurately probes and records the surface profile on five 3-mm-long sections of each specimen [14].

Atomic force microscopy (AFM): AFM is used to measure the surface roughness of materials by profilometric or optical methods and is generally expressed as root mean square (RMS) values.This technique can provide detailed information about the surface topography and roughness of archwires. 3D optical profilometry: This method uses white light that passes through a beam filter, directing the light to the sample surface and a reference mirror. When the light reflected from these two surfaces recombine, a pattern of interference arises, and from these, surface roughness is determined. Energy-dispersive X-ray analysis (EDX): This technique is used to analyze the chemical composition of the wire surface by determining the elemental composition of a material on interaction with X-rays, depending on the energy differences that occur during excitation and downfall of its electrons. Raman spectroscopy: Raman spectroscopy is based on the in-elastic scattering of a monochromatic laser with a material and can be used to reconfirm the surface composition of each group. These methods can be used individually or in combination to provide a comprehensive assessment of the surface structure analysis of archwires [15].

A Fractal dimension (FD): analysis is used in the estimation of complex, irregular shapes or surfaces. As the fractal architecture concept is particularly interesting in surface and material science, it can also be adapted to assess the surface of titanium endosseous implants. Fractal dimension analysis (FDA) has been reported in the surface testing of various dental materials, among them xenogenic bone, lithium disilicate-based crowns, zirconia dental implants, or dental restorative, composite, orthodontic wires. Texture analysis (TA): is another mathematical method that enables the analysis of the surface. The pixels form an image structure called a texture. A texture is a set of graphic patterns which are characterized by brightness, entropy, smoothness, uniformity, roughness, granularity, randomness, or linearity [16].

SEM (Scanning Electron Microscopy) has several advantages over fractal dimension and texture analysis in the surface analysis of archwires. Firstly, SEM provides high-resolution images, which can reveal more detailed surface features compared to fractal dimension and texture analysis. SEM can capture images with a resolution of up to 1 nm, allowing for a more precise analysis of the surface structure. Secondly, SEM allows for the visualization of the 3D surface topography, which can provide more comprehensive information about the surface properties than fractal dimension and texture analysis. SEM can reveal features such as roughness, porosity, and surface defects, which are crucial for understanding the mechanical properties of archwires. Thirdly, SEM can be used to analyze the surface chemistry of archwires. SEM can be combined with other techniques, such as Energy Dispersive X-ray Spectroscopy (EDS), to determine the elemental composition of the surface. This information can be essential for understanding the corrosion resistance and biocompatibility of archwires. Fourthly, SEM can be used to analyze the surface roughness of archwires. SEM can provide information about the height, width, and density of surface roughness, which can be used to predict the friction between the archwire and the bracket. This information is crucial for optimizing the orthodontic treatment plan. Lastly, SEM is a versatile technique that can be used to analyze a wide range of materials, including metals, ceramics, and polymers. This versatility makes SEM an ideal tool for the surface analysis of archwires, which are typically made of various materials [17].

The mechanical properties of orthodontic wires are crucial for their effectiveness in correcting malocclusions. The properties required for orthodontic wires depend on their application, but commonly desirable mechanical characteristics include high spring back, low stiffness, good formability, and low friction. Different wires made from different metals and alloys are available in the market, and they must obey certain properties such as biocompatibility, formability, weldability, low coefficient of friction, resilience, shape memory, low stiffness, and high elastic limit. However, the oral cavity’s environment can significantly affect the mechanical properties of orthodontic wires. For example, the oral cavity’s temperature, humidity, and pH can affect the mechanical properties of orthodontic wires, leading to changes in their elasticity, strength, and surface characteristics [18].

In addition, the oral cavity’s microbial flora can also affect the mechanical properties of orthodontic wires. Bacterial colonization on the surface of orthodontic wires can lead to the formation of biofilms, which can alter the wires’ surface properties and increase their susceptibility to corrosion. Moreover, the oral cavity’s dietary habits, such as the consumption of acidic or sugary foods and drinks, can also affect the mechanical properties of orthodontic wires. For example, the immersion of orthodontic wires in carbonated drinks can lead to a decrease in their mechanical properties, such as their elastic modulus and ultimate tensile strength. Furthermore, the oral cavity’s mechanical forces, such as those generated by bruxism or other parafunctional habits, can also affect the mechanical properties of orthodontic wires. These forces can lead to the deformation or fracture of the wires, reducing their effectiveness in correcting malocclusions. Therefore, it is essential to consider the oral cavity’s environment when selecting and using orthodontic wires [18].

Using archwires for durations more significant than one month is typical during more advanced phases of orthodontic therapy. Our 30-day analysis of the corrosion of NiTi archwires with various types of mouthwash revealed significant variations.

### Corrosion

In the study of the control group, the corrosion of NiTi archwires was greater anteriorly than posteriorly. At the 5% probability level, differences between these two parts of both archwires were not statistically significant [19, 20]. But others reported a significant difference between these two parts of both archwires [21, 22]. The discrepancy in previous research findings may be partially attributable to the fact that these studies were conducted in artificial saliva at constant pH level. In this study, NiTi archwires were placed in patient mouths with variable pH, resulting in the destruction of the archwire’s protective layer under low pH conditions.

In the sodium fluoride and chlorhexidine group, corrosion occurred anteriorly more frequently than posteriorly. The differences between anterior and posterior parts for NiTi wires were statistically highly significant at the 1% probability level. This may be because the mouthwash has a more significant impact on the anterior part of the archwire, where it is diluted by saliva. Meanwhile, a highly significant difference was found between NiTi wires in the anterior and posterior parts.

The sodium fluoride mouthwash caused higher corrosion than the control group and the chlorhexidine mouthwash. This result may be attributed to the high affinity of titanium to hydrogen [23]. In fluoride solutions, a reaction between the protecting titanium film and hydrofluoric acid takes place to form sodium titanium fluoride, which destroys the protective film. The fluoride ion might not prevent the formation of oxide layers on the electrode surfaces. However, it may affect the characteristics of the oxide layer [24]. Consequently, the corrosion resistance of titanium alloys decreases in solutions having fluoride [25–31].

In the chlorhexidine group, a statistically non-significant difference was found between NiTi archwires in the anterior part. However, a highly significant difference was found between them in the posterior part [20, 22]. In contrast, the SEM of NiTi archwires show scratches and fissures (Fig. 1), which may be associated with the pattern of surface flaws formed during the wire manufacturing process. These defects are the preferential corrosion areas [32]. These defects were more prominent in NaF than in CHX mouthwash. This may be due to the fact that the oxide film of NiTi archwire dissolves more quickly in fluoride solution than in chloride solution, and the reduction in corrosion resistance is more pronounced [33].

A statistically significant difference was found between the three groups in the anterior section. However, a statistically significant difference was found between the control and the two other groups in the posterior section. In addition, a non-statistically significant difference was found between the NaF and CHX groups, as both induced a higher corrosion rate than the control group. The overall *p*-value of both parts of the comparison between groups (control, NaF, and CHX) was statistically significant, particularly between the control group and NaF [20, 31]. But others reported not statistically significant, particularly between the control group and NaF [8]. These contradictory findings could be due to the difference in materials and methodology.

According to this study’s results, the corrosion rate was higher in nickel-titanium archwires, and sodium fluoride mouthwash produced more corrosion than chlorhexidine mouthwash.

**Limitations of the study and future directions**.

- It should be also noted that the frictional forces recorded in this study were substantially different from the actual applied forces in orthodontic movement.
- Many intraoral variables such as saliva, plaque, chewing, bone density, tooth number, anatomic configuration, and occlusion can influence frictional force levels, and were not evaluated in the present study.
- Another limitation of this study is that other types of orthodontic wires such as beta titanium wires have not been investigated, and thus, the obtained results cannot be extrapolated to them.
- The surface structure should also be better analyzed with advanced equipment, i.e., atomic force microscopy (AFC) so that we could measure the quantity of the surface by its roughness. This will allow us to better understand the physical properties of archwires.

## Conclusions

Within the drawbacks of the current study, the following could be concluded:

1. Changes in the surface due to corrosion of NiTi archwires were significantly in control and mouthwash groups.
2. Changes in the surface of NiTi archwires by NaF mouthwashes were higher than control and CHX groups.
3. The mouthwash has to be recommended in accordance with the orthodontic materials used to decrease the adverse effects of various mouthwashes. However, chlorhexidine-containing mouthwash might be the mouthwashes of choice during all phases of orthodontic treatment.
4. Therefore, these prophylactic agents should be used with caution, and new mouthwashes are required that have less detrimental effects on corrosion and, consequently, the mechanical characteristics of orthodontic archwires.
5. During the initial stage of orthodontic biomechanics, it is advised to use archwires that may deliver light, long-lasting, and stable force values. These wires should also be resistant to corrosion induced by anticorrosion agents.

**Fig. 2:**
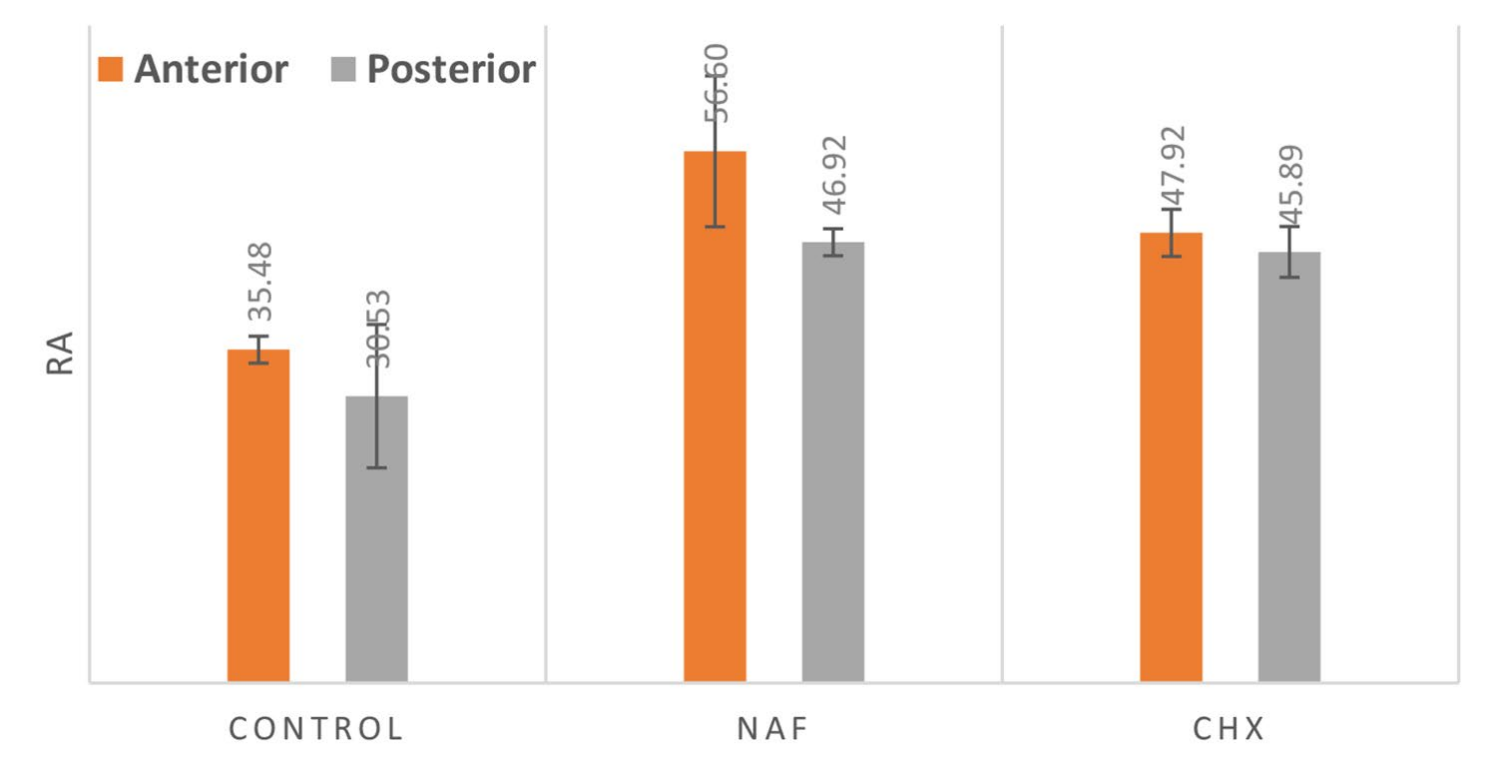
Bar chart presenting the surface roughness between different parts of NiTi archwire in all groups

## Data Availability

Upon a reasonable request, the corresponding author can provide the datasets utilized and/or generated in the current research.

## Abbreviations

***SEM***: Scanning electron microscopy
***CHx***: Chlorhexidine
***NaF***: Sodium Fluoride
***NiTi***: Nickel Titanium
***SD***: Standard deviation
***BSE***: Backscattered electron
SPSS: Statistical Package for Social Science
Cr: Chromium
Ra: Arithmetic mean roughness
